# P-1520. Immune Depression Associated with Viral DNAemia in Pediatric Sepsis

**DOI:** 10.1093/ofid/ofaf695.1704

**Published:** 2026-01-11

**Authors:** Zachary Aldewereld, Brendan Connolly, Joe Carcillo

**Affiliations:** Univ of Pittsburgh / UPMC Children's Hospital of Pittsburgh, Pittsburgh, Pennsylvania; University of Michigan, Ann Arbor, Michigan; Univ of Pittsburgh / UPMC Children's Hospital of Pittsburgh, Pittsburgh, Pennsylvania

## Abstract

**Background:**

Sepsis remains a significant cause of pediatric mortality and morbidity. We recently reported that viral DNAemia was associated with increased mortality in pediatric sepsis, and that persistent lymphopenia early in the course of sepsis was independently associated with subsequent development of secondary infection. This study aims to further understand the immunologic alterations associated with these findings.

**Figure:**
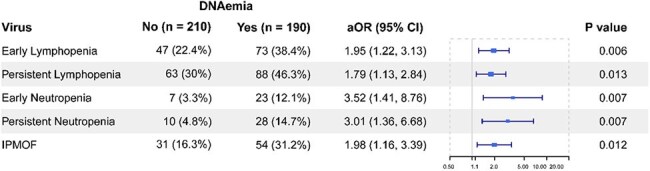
Adjusted association of viral DNAemia with early markers of immune depression Patients with viral DNAemia during their course were more likely to have multiple markers of immune depression after adjusting for age, max organ failure index on day 0, PRISM, and immunocompromised status. Persistent lymphopenia was also independently associated with secondary infection in prior analyses. IPMOF: Immunoparalysis-associated multiorgan failure.

**Figure:**
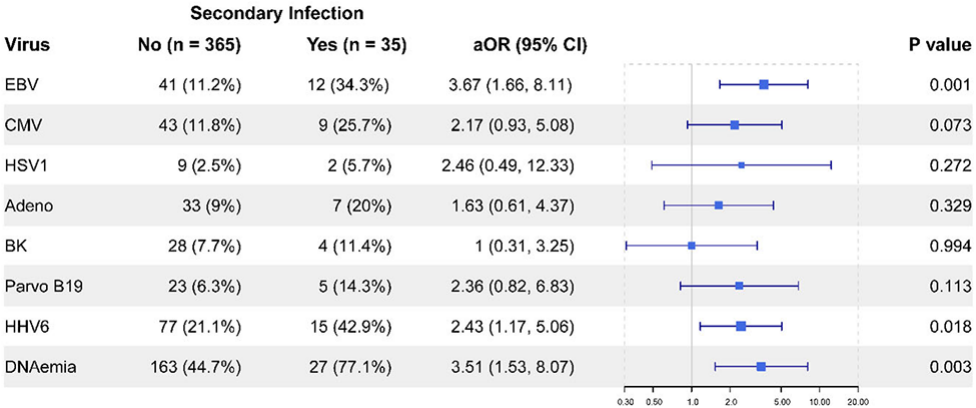
Adjusted association of secondary infection with viral DNAemias After adjusting for persistent lymphopenia and persistent neutropenia (independently associated with secondary infection in all patients and immunocompromised patients, respectively), patients with secondary infection were more likely to have had EBV DNAemia, HHV6 DNAemia, or DNAemia of any of the listed viruses.

**Methods:**

Secondary analysis of 401 pediatric sepsis patients from 9 Pediatric Intensive Care Units. Samples were tested by PCR for EBV, CMV, HSV, adenovirus, HHV6, BKV, and parvovirus B19. Culture information was recorded daily for the first 28 days. Associations between DNAemia, immune depressions markers, and stringently defined secondary infection were assessed with multivariable logistic regression.

**Figure:**
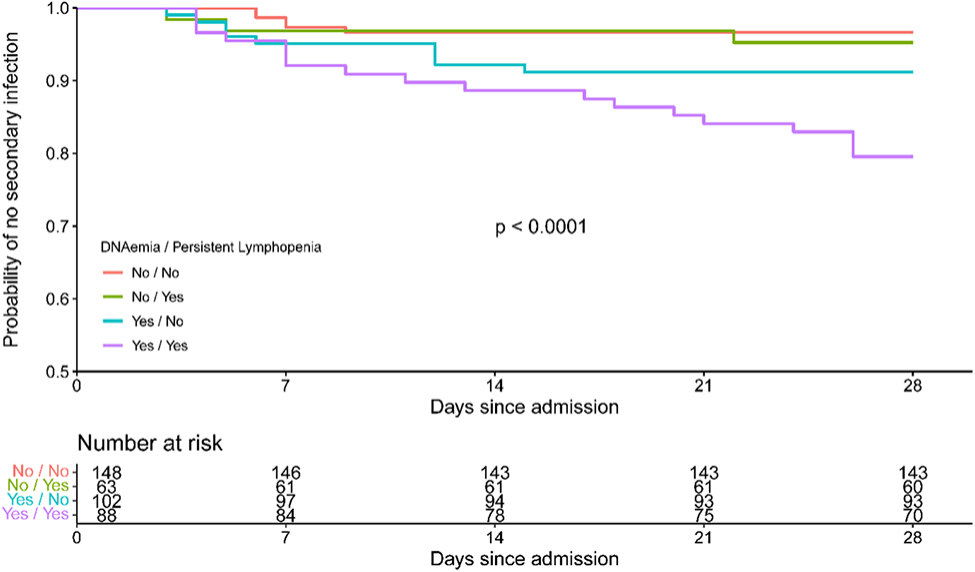
Time to secondary infection by DNAemia and persistent lymphopenia status Patients with both DNAemia and persistent lymphopenia were most likely to develop secondary infection during their course.

**Results:**

Patients with viral DNAemia during their course were more likely to have had early lymphopenia (aOR 1.95, p=0.006), persistent lymphopenia (aOR 1.79, p=0.013), early neutropenia (aOR 3.52, p=0.007), or persistent neutropenia (aOR 3.01, p=0.007) after adjusting for age, max organ failure index on day 0, PRISM, and immunocompromised status (Fig 1). Patients who developed secondary infections of any type were more likely to have had DNAemia (77% vs 45%, aOR 3.5, p=0.003) after adjusting for persistent lymphopenia and persistent neutropenia (Fig 2). Similar findings were seen for EBV (34% vs 11%, aOR 3.7, p=0.001) and HHV6 (43% vs 21%, aOR 2.4, p=0.018). Risk of secondary infection was greatest in those who had both DNAemia and persistent lymphopenia (Fig 3). The largest proportion of secondary infections was viral in both groups (No DNAemia: 5/8, 62%; DNAemia: 12/27, 48%) followed by bacterial (3/8, 38%; 9/27, 33%) and fungal (0, 0%, 5/27, 19%), and this was not significantly different in those who did or did not have DNAemia (p=0.56).

**Conclusion:**

Patients who were found to have DNAemia demonstrated multiple markers of immune depression and were more likely to develop secondary infections. Viral DNAemia is associated with immune derangements in sepsis and are likely indicative of an immune depressed state.

**Disclosures:**

All Authors: No reported disclosures

